# Interleukin-8 gene polymorphism –251T*>*A contributes to Alzheimer's disease susceptibility

**DOI:** 10.1097/MD.0000000000005039

**Published:** 2016-09-30

**Authors:** Biyong Qin, Li Li, Shanshan Wang, Jun Wu, Yulan Huang, Ping Zhou, Jiao Bai, Yan Zheng

**Affiliations:** a Department of Neurology; b Department of Nursing; c Department of Public Health; d Department of Cardiology; e Department of Spinal Surgery; f Department of Ultrasonography, Renmin Hospital, Hubei University of Medicine, Shiyan, Hubei, China.

**Keywords:** Alzheimer's disease, interleukin-8, meta-analysis, polymorphism

## Abstract

**Background:**

Published association studies have investigated the correlation between interleukin-8 (IL-8) gene polymorphism –251T>A and susceptibility to Alzheimer's disease (AD); however, the results are conflicting. Thus, we conducted the meta-analysis to reassess the effect of IL-8 gene –251T>A variant on the risk of AD.

**Methods:**

Relevant studies regarding this association were electronically searched and identified from the PubMed, Embase, Cochrane Library, China National Knowledge Infrastructure, and the Chinese Biomedicine Database. The odds ratios (ORs) with the corresponding 95% confidence intervals (95% CIs) were pooled to calculate the strength of this association.

**Results:**

Nine studies with a total of 1406 cases and 2152 controls were included in the meta-analysis. Overall, a significant association of IL-8 gene –251T>A polymorphism with increased risk of AD was observed in several genetic models (allele, A vs T: OR=1.32, 95%CI=1.16-1.50; homozygous, AA vs TT: OR=1.70, 95%CI=1.21–2.21; heterozygous, TA vs TT: OR=1.37, 95%CI=1.12–1.69; recessive, AA vs TA+TT: OR=1.40, 95%CI=1.12–1.75). Similarly, such association was also revealed both in Asian and European populations in the subgroup analysis by ethnicity.

**Conclusion:**

The current study suggested that IL-8 gene polymorphism –251T>A may contribute to the susceptibility to AD.

## 1 Introduction

Alzheimer's disease (AD) posting the major global cause of dementia affecting seriously the quality of life has represented a large burden on the health of the elderly, with the increase of life spans and the number of elderly patients in the coming years.^[1]^ Therefore, considerable efforts to the understanding of the etiology of AD toward early detection and effective treatment are of great importance. A growing body of evidence has shown that AD is a progressive neurodegenerative disorder characterized by the loss of memory, mental confusion, and several cognitive disturbances.^[2]^ Numerous studies have proven that the chronic inflammation plays a vital role in the pathogenesis of AD,^[3]^ which involved 2 typical neuropathological alterations in the brain of patients with AD: amyloid plaques and neurofibrillary tangles accompanied with amyloid-beta peptide (Aβ) deposition and tau protein aggregation, which results in neurons damage.^[4]^ Recently, a variety of inflammatory cytokines/chemokines, including interleukin-8 (IL-8), IL-6, IL-1, tumor necrosis factor-alpha (TNF-α), and monocyte chemotactic protein 1 (MCP-1), were detected with a markedly elevated level in patients with AD, supporting the importance of cytokines/chemokines in the development of AD.^[5–8]^

IL-8 as a CXC chemokine is located on chromosome 4q13–21 consisting of 4 exons, 3 introns, and a proximal promoter region, and usually served as a chemoattractant of neutrophils and lymphocytes in inflammatory activity.^[9]^ The concentration of IL-8 in cerebrospinal fluid and postmortem adult human microglia of patients with AD were significantly increased, suggesting the importance of IL-8 upregulation in the AD pathogenesis.^[10]^ Although the role of IL-8 in the development of AD remained largely unknown, there was a possibility for the underlying mechanism implied by the previous investigations that the abnormal deposition of Aβ stimulated by immunogens in AD patients was able to lead to the activation of microglia and release of inflammatory cytokines including IL-8, and subsequently result in neurons damage through the direct or indirect toxic effects of chronic inflammatory response.^[5,8]^ During the process of inflammatory response, IL-8 chemokine activity has been found to have a momentous effect on the enhancement of Aβ-induced proinflammatory responses mediated by activated microglia in the AD brain.^[11,12]^

Individual differences in the production of IL-8 have been linked to a functional polymorphism (T>A at position –251) in the promoter region of IL-8 gene and the AA genotype was reported to contribute most to the expression and activity of IL-8 chemokine.^[12,13]^ Given these findings, several lines of evidence have revealed a significant association of IL-8 gene –251T>A with altered risk of AD, however, obtained conflicting results, which may be due to the ethnicity variation and relatively small size of individual study. To derive a more precise estimation on the association between IL-8 gene –251T>A polymorphism and susceptibility to AD, a meta-analysis was carried out with all available case-control studies as yet.

## 2 Methods

### 2.1 Literature search

An overall electronic literature search in PubMed, Embase, Cochrane Library, China National Knowledge Infrastructure, and the Chinese Biomedicine Database was undertaken for all the potentially relevant studies focusing on the association between IL-8 gene –251T>A polymorphism and susceptibility to AD up to September 2015. The language was restricted to English and Chinese in the literature search. The search strategy was developed by variable combinations with the following query: [“Interleukin-8” or “IL-8”] and [“Alzheimer's disease” or “AD”] and [“polymorphisms” or “polymorphism” or “variant” or “genotype”]. The references of all identified publications and relevant reviews were individually and manually searched for additional studies. Ethical approval and patient written informed consent were not required owing to that this was a meta-analysis of previously published studies.

### 2.2 Criteria for inclusion and exclusion

Studies were included if they satisfied the following inclusion criteria: (1) case-control studies investigating the association between IL-8 gene –251T>A polymorphism and susceptibility to AD; (2) sufficient information offered for calculating odds ratios (ORs) and 95% confidence intervals (95%CIs); (3) original data available to be abstracted for the frequencies of alleles or genotypes in case and control groups; (4) studies definitely describing the diagnostic criteria for AD. Major exclusion criteria were: (1) studies without control population; (2) family-based studies; (3) duplication of the previous study with a smaller sample size; (4) abstracts, comments, reviews, or editorial articles lack of necessary raw data.

### 2.3 Data extraction

The following information from identified eligible studies was independently extracted by 2 investigators with a uniformed layout: first author, publication year, study country, ethnicity, numbers of case and control, genotyping methods, frequencies of genotype in case and control, source of control, and evidence of Hardy–Weinberg equilibrium (HWE). Ethnic groups were classified as Asians and Europeans. Any discrepancy was resolved by discussion and consultation with a third reviewer if necessary.

### 2.4 Statistical analysis

In light of the previous findings that IL-8 gene –251T>A polymorphism was stated to affect the expression and activity of IL-8 chemokine, we analyzed the data for dominant (AA+TA vs TT), allele (A vs T), homozygous (AA vs TT), heterozygous (TA vs TT) and recessive (AA vs TT+TA), and hypothesized that this polymorphism is a risk factor for AD. The pooled ORs and 95% CIs were calculated to assess the associations between IL-8 gene –251T>A polymorphism and the risk of Alzheimer's disease. The significance of the pooled OR was determined by *Z* test (*P* < 0.05 was considered significant) using Review Manager version 5.2 software (provided by The Cochrane Collaboration, Oxford, UK; http://www.cochrane.org/software/revman.htm). The heterogeneity across included individual studies was assessed by both *Q* statistic test and *I*^2^ test, and *P* < 0.1 and *I*^2^>50% indicated the obvious existence of heterogeneity.^[14]^ A random-effect model (DerSimonian–Laird method) was applied to calculate the pooled ORs in the case of the presence of heterogeneity;^[15]^ otherwise, a fixed-effect model (Mantel–Haenszel method) was selected.^[16]^ Stratified analyses were conducted by ethnicity. Begg's funnel plot test and Egger's regression test were utilized to assess the publication bias using Stata 12.0 software (Stata Corp., College Station),^[17]^ and *P* < 0.05 was considered significant.

## 3 Results

### 3.1 Study selection and characteristics of included studies

**Figure 1:**
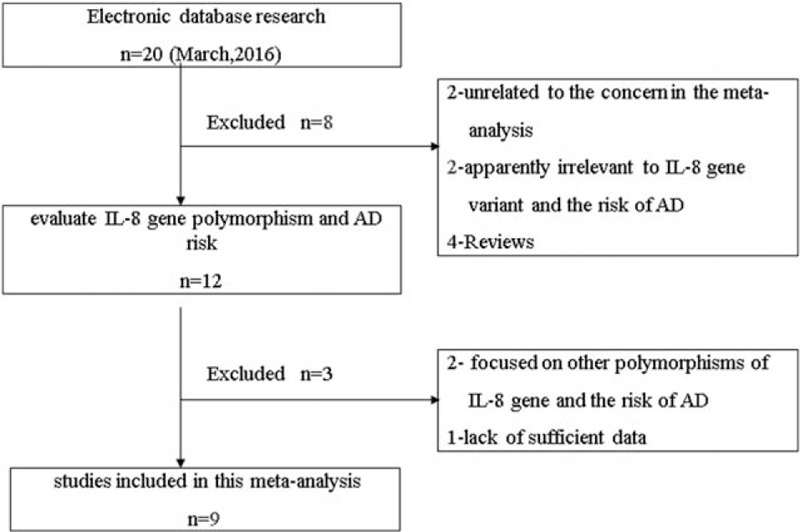
Flowchart of study selection procedure.

In total, 20 potentially relevant papers were retrieved by the initial search according to the established search strategy. After a further review, 11 studies were excluded due to the following reasons: 2 articles identified as reviews were first excluded, 2 articles were excluded due to the topic apparently irrelevant to the concern in the current study, 4 articles were also excluded because these studies focused on the association of AD risk and polymorphisms of other genes, 2 articles were excluded for that they concerned the relationship between other polymorphisms of IL-8 gene and AD risk, and 1 article was excluded because of the lack of sufficient data of genotype distribution. Finally, a total of 9 case-control studies with 1406 cases and 2152 controls were included to investigate the association of IL-8 gene –251T>A polymorphism and susceptibility to AD in the meta-analysis.^[2,12,18–24]^ Of which, there were 4 studies conducted in Asians and 5 studies in Europeans. The frequencies of genotypes in controls of all included studies were in agreement with HWE except for the study by Li et al.^[12]^ Moreover, as only the frequency of AA+TA genotype was provided in the studies of Combarros et al^[23]^ and Infante et al,^[24]^ the 2 studies were pooled into the meta-analysis only in the dominant model. The flowchart of study selection procedure is shown in Fig. 1 and detailed characteristics of the included studies are presented in Table 1.

**Table 1:**
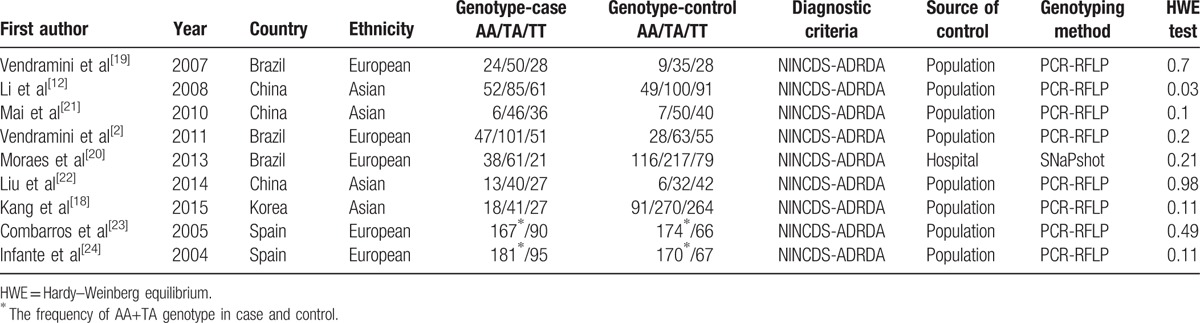
Characteristics of included studies in the meta-analysis.

### 3.2 Meta-analysis results

**Figure 2:**
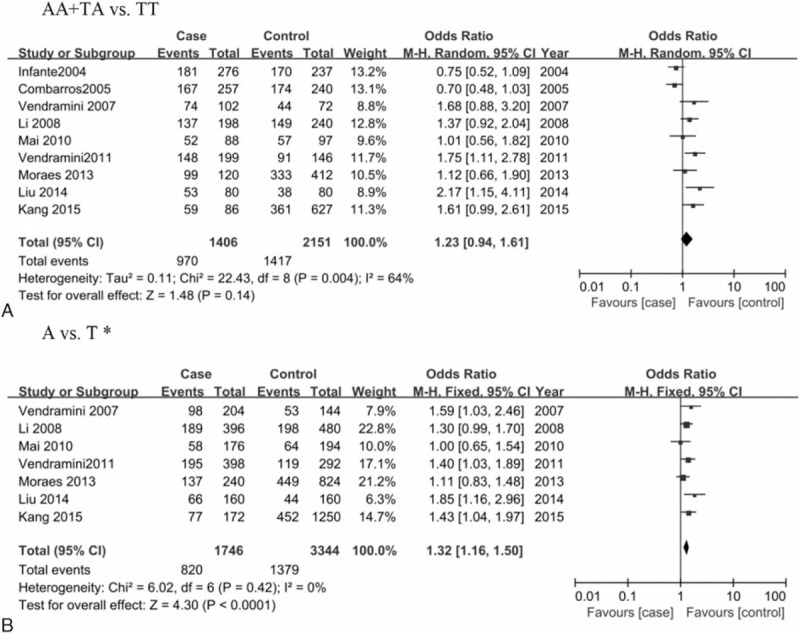
Forest plot for the association between IL-8 gene polymorphism –251T>A and AD risk in the dominant model. ∗, as only the frequency of AA+TA genotype was provided in the studies of Combarros et al^[23]^ and Infante et al^[24]^, the 2 studies were pooled into the meta-analysis only in the dominant model. AD = Alzheimer's disease.

As shown in Table 2 and Fig. 2, no obvious evidence of heterogeneity was detected in 5 genetic models (all *P* > 0.1) except for the dominant model, an appropriate effect model based on the heterogeneity was thereby selected for the pooled analyses. Overall, a significant association of IL-8 gene –251T>A polymorphism with increased risk of AD was observed (allele, A vs T: OR = 1.32, 95%CI = 1.16–1.50; homozygous, AA vs TT: OR = 1.70, 95%CI = 1.21–2.21; heterozygous, TA vs TT: OR = 1.37, 95%CI = 1.12–1.69; recessive, AA vs TA+TT: OR = 1.40, 95%CI = 1.12–1.75). Similarly, such association was also revealed both in Asian and European populations in the subgroup analysis by ethnicity. The main results of meta-analysis are summarized in Table 2.

**Table 2:**
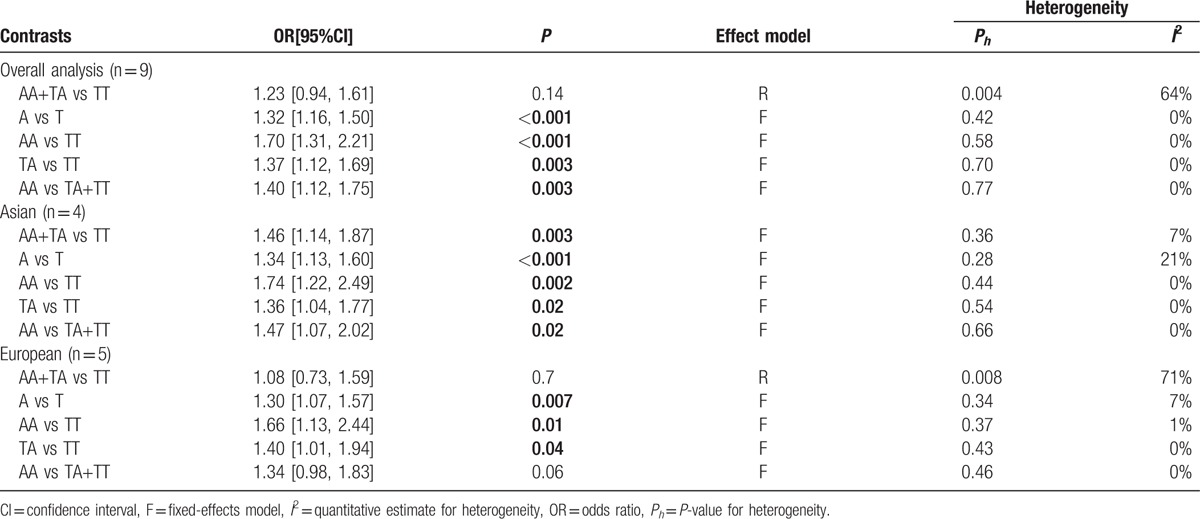
Meta-analysis results for the association of IL-8 gene –251T>A polymorphism with the risk of Alzheimer's disease.

### 3.3 Sensitivity analysis and publication bias

**Figure 3:**
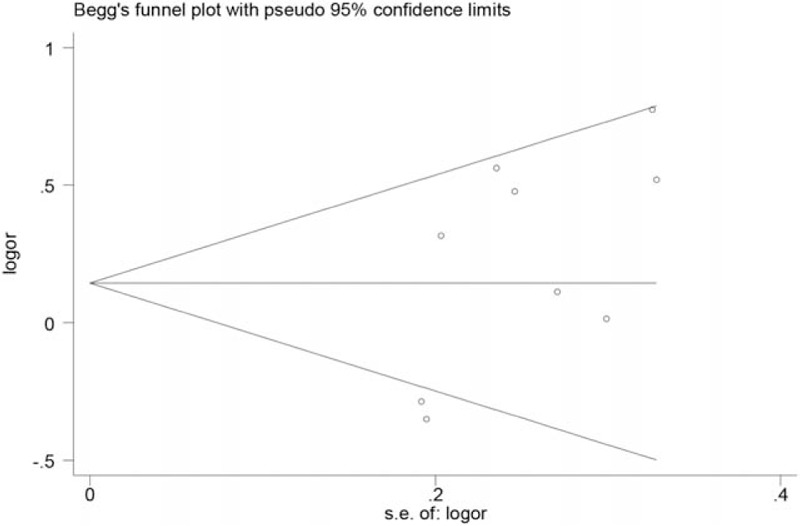
Begg's funnel plot for publication bias in the dominant model.

Sensitivity analysis was performed by sequential removal of the individual study and the results showed no significant alteration of pooled ORs in all genetic models except for the exclusion of studies by Combarros et al^[23]^(pooled OR=1.32, 95%CI=1.03–1.71, *P*=0.03) or Infante et al^[24]^ (pooled OR=1.32, 95%CI=1.00–1.73, *P* = 0.05) in the dominant model, suggesting that the significance of the polymorphism may be masked by the considerable heterogeneity across studies. Publication bias was assessed by Begg's funnel plot test and Egger's test. As shown in Fig. 3, no obvious asymmetry was observed on the funnel plot, suggesting no evidence of publication bias, which was also supported by Egger's test (AA+TA vs TT: *P* = 0.075, data for other genetic model not shown).

## 4 Discussion

The present study is the first to evaluate the association between IL-8 gene polymorphism –251T>A and AD risk with a quantitative approach, representing the largest study so far. The results of the meta-analysis provided strong evidence demonstrating a significant association of IL-8 gene –251T>A variant with an increased risk of AD.

Accumulative evidence has demonstrated the vital role of IL-8 in most inflammation-related disorders, including AD. Mechanistically, the further exploration suggested that IL-8 might serve as an important mediator of neuronal death in AD both via its effects on release of neurotoxins such as MMP-2 and MMP-9 as well as by induction of cell cycle protein cyclinD1 and pro-apoptotic protein Bim.^[25]^ To date, although the pathogenesis of AD was not fully understood due to the complexity of the etiology, the critical role of genetic components in the development and progression of AD has been firmly confirmed by evidences.^[2,26,27]^ A functional polymorphism –251T>A in the proximal promoter region of IL-8 gene has been ever found to be associated with several inflammatory diseases, such as bronchial asthma,^[28]^ gastritis,^[29]^ and pancreatitis,^[30]^ which may be due to the frequently reported relationship between –251A allele and higher IL-8 transcription activity, thereby which is able to result in altered susceptibility to diseases.^[31]^ Likewise, studies regarding the association of IL-8 gene polymorphism –251T>A with susceptibility to AD in populations have emerged in recent years, however, obtained discrepant results. Mai et al^[21]^ and Kang et al^[18]^ found an overall significant association between the IL-8 gene –251T>A variant and increased individual susceptibility to AD, whereas other studies failed to reveal such association,^[20,22,24]^ which prompted the further investigations to verify the association with a large-scare sample size.

In the current meta-analysis, we found evidence of a significant association between the IL-8 gene –251T>A polymorphism and increased risk of AD by the overall analysis with 9 case-control studies involving 1406 cases and 2152 controls, which was not in line with some individual studies, indicating that the sample size was likely significant for the assessment concerning the IL-8 gene –251T>A polymorphism and the risk of AD. In the stratification analysis based on ethnicity, a significantly increased risk of AD associated with IL-8 gene –251T>A variant was revealed among Asians in all 5 genetic models and Europeans in 3 genetic models, suggesting that the ethnicity variation might contribute less to this association. Nevertheless, this association should be further validated in more populations, especially Europeans due to the limited number of included studies and obvious heterogeneity and there was a lack of Africans involved in the association, which would restrict the comprehensiveness and representativeness of the results.

Despite these interesting results, several possible limitations of this meta-analysis should be acknowledged. First, though there was no obvious publication bias suggested in present meta-analysis, the possibility of publication bias were inevitable because studies with positive results were easily to be published in general, while studies with negative findings may not have been published. Second, the data from included studies were primarily based unadjusted estimates due to the lack of original information, some important compounding factors such as age, gender, and education should be taken into consideration. Third, AD is a complex disease that may result from the combined effects of multifactor, including gene–gene interactions. For example, the interactions between a well-known risk factor of AD risk apolipoprotein E gene ε4 and IL-8 gene locus, IL-8 gene –251T>A, and IL-1α gene -899C>T variants have been reported to influence the susceptibility to AD.^[24]^ Additionally, gene–environment interactions may be involved critically in the development of AD, which should be considered for the association as well. Nevertheless, relatively less heterogeneity and no obvious publication bias together suggested the reliability and robustness of the results in the meta-analysis, which was of great importance for a quantitative approach.

## 5 Conclusion

In conclusion, the current study suggested that IL-8 gene polymorphism –251T>A was associated with an increased risk of AD. Large well-designed studies with diverse populations and functional evaluations concerning this association are warranted to confirm and extend our findings.
